# Successful Treatment of Verrucous Epidermal Nevus With Full-Thickness Skin Excision: A Case Report and Literature Review

**DOI:** 10.7759/cureus.91557

**Published:** 2025-09-03

**Authors:** Ricardo Cid-Puente, Frida I Rosas-Lezama, Laura S De León-Puga, Amairani Tovar-Garcia, Julieta C Corral-Chavez

**Affiliations:** 1 Department of Immunology, School of Biological Sciences, Universidad Autónoma de Zacatecas, Zacatecas, MEX; 2 Department of Internal Medicine, Hospital General Regional No.12 "Lic. Benito Juarez Garcia" Instituto Mexicano del Seguro Social, Merida, MEX; 3 Department of Internal Medicine, Hospital Regional Pemex, Ciudad Madero, MEX; 4 Department of Internal Medicine, Hospital General de Xoco, Secretaria de Salud, Mexico City, MEX; 5 Dermatopathology, Private Practice, Fresnillo, MEX

**Keywords:** blaschko’s lines, congenital nevus, epidermal nevus, surgical skin excision, verrucous epidermal nevus

## Abstract

Verrucous epidermal nevus (VEN) is a benign congenital hamartoma arising from abnormal keratinocyte proliferation, often presenting as linear, hyperpigmented, verrucous plaques along the lines of Blaschko, and it is usually a cause of major cosmetic concern. We present the case of an 18-year-old woman with a VEN on the medial aspect of her left arm, progressively enlarging since infancy. Examination revealed a 5 cm × 2 cm × 1 cm linear, hyperpigmented, verrucous plaque. A complete surgical excision with 3-mm margins was performed and histopathology confirmed the diagnosis of VEN. At two-month follow-up, the patient had complete wound healing, no recurrence, and satisfaction with the cosmetic result. This case highlights that complete surgical excision remains the gold standard for localized VEN, offering definitive cure and excellent long-term outcomes for appropriately selected patients, despite the risk of scarring.

## Introduction

Verrucous epidermal nevi (VEN) are benign congenital skin lesions defined as hamartomas, which result from an abnormal proliferation of keratinocytes. These lesions characteristically present as linear, hyperpigmented, and warty-textured (verrucous) plaques that follow the lines of Blaschko on the skin [1]. VENs are rooted in genetic mosaicism, arising from postzygotic somatic mutations in genes that regulate the growth of epidermal cells [2].

Typically, these lesions are present at birth or become apparent in early childhood, often growing in size and changing in color over time. While many are isolated findings that pose primarily a cosmetic concern, they can sometimes be associated with systemic conditions affecting the neurologic, skeletal, or ocular systems, which are collectively known as epidermal nevus syndromes (ENS). Additionally, though uncommon, there is a risk of malignant transformation into skin cancers like basal cell or squamous cell carcinoma within long-standing lesions [1,3].

The management of VEN can be challenging, as treatments range from topical agents with modest results to various ablative techniques and surgical excision. Complete surgical removal is considered the most definitive curative approach, but it results in scarring [4]. This report presents the case of an 18-year-old woman with an isolated VEN, detailing its clinical presentation, the rationale for surgical management, and the successful outcome, followed by a review of the current literature on the topic.

## Case presentation

An 18-year-old female university student, with no significant past medical or family history, presented for evaluation of a lesion on the medial aspect of her left arm that had become a cosmetic concern. According to the patient's history, the lesion had been present since the first few months of life. It initially appeared as a small, skin-colored, verrucous papule, approximately 3 mm long. Over the years, it had progressively enlarged in both length and height while developing a dark brown hyperpigmentation.

**Figure 1:**
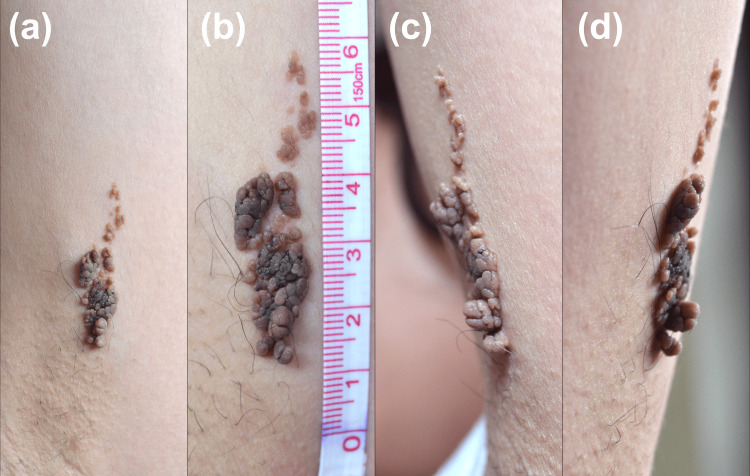
Verrucous epidermal nevus Macroscopic view of the lesion. (a) Frontal aspect of the left arm just above the armpit. (b) Close up view of the lesion. (c) Left aspect of the left arm. (d) Right aspect of the left arm.

On physical examination, a linear, hyperpigmented, and verrucous plaque was noted on the medial aspect of the left arm, following the lines of Blaschko. The lesion measured 5 cm in length, 2 cm in width, and 1 cm in height. It was non-tender upon palpation, and the rest of the cutaneous examination was unremarkable (Figure 1).

**Figure 2:**
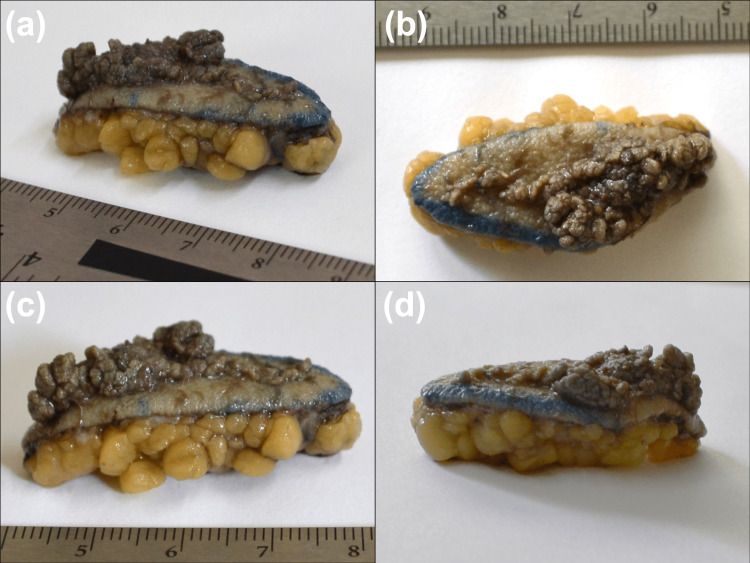
Verrucous epidermal nevus (VEN) biopsy The image presents a full-thickness skin biopsy of a VEN measuring 4 cm in length and 2 cm in width. (a) Oblique view, (b) upper view, and (c,d) lateral view are shown.

**Figure 3:**
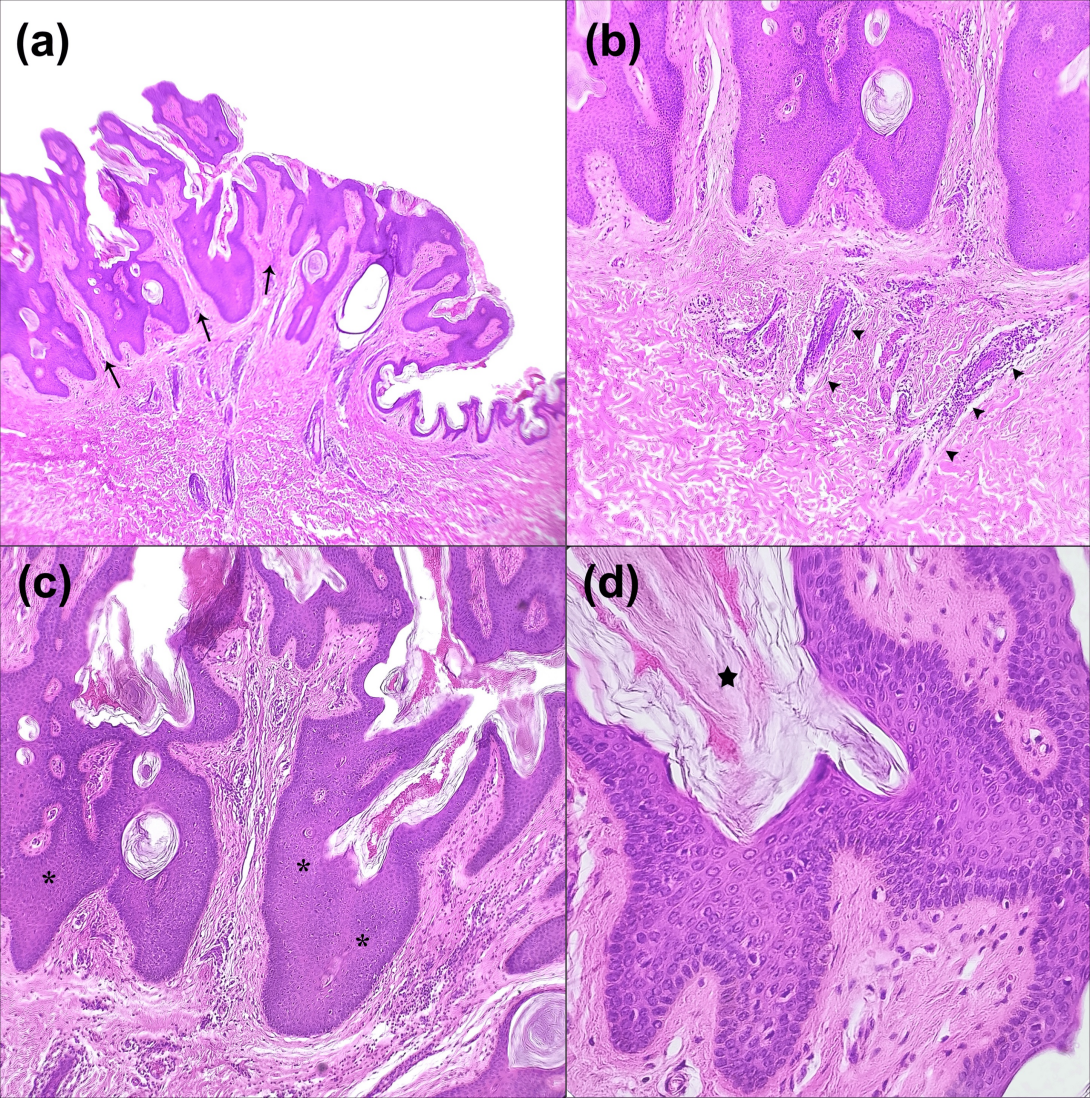
Histopathologic findings Histopathological examination with hematoxylin and eosin staining. (a) Low-power magnification (4×) demonstrates epithelial proliferation characterized by pronounced papillomatosis with prominent vertical projections (black arrows), focal irregular hypergranulosis, and basal layer hyperpigmentation. (b) Low-power view (4×) reveals papillary and superficial reticular dermis with elongated interpapillary processes and mild perivascular lymphocytic infiltrate (black arrowheads). (c) Intermediate magnification (10×) illustrates irregular acanthosis of the stratified squamous epithelium (black asterisk). (d) High-power magnification (40×) exhibits the stratum corneum with prominent lamellar hyperkeratosis (black star).

**Figure 4:**
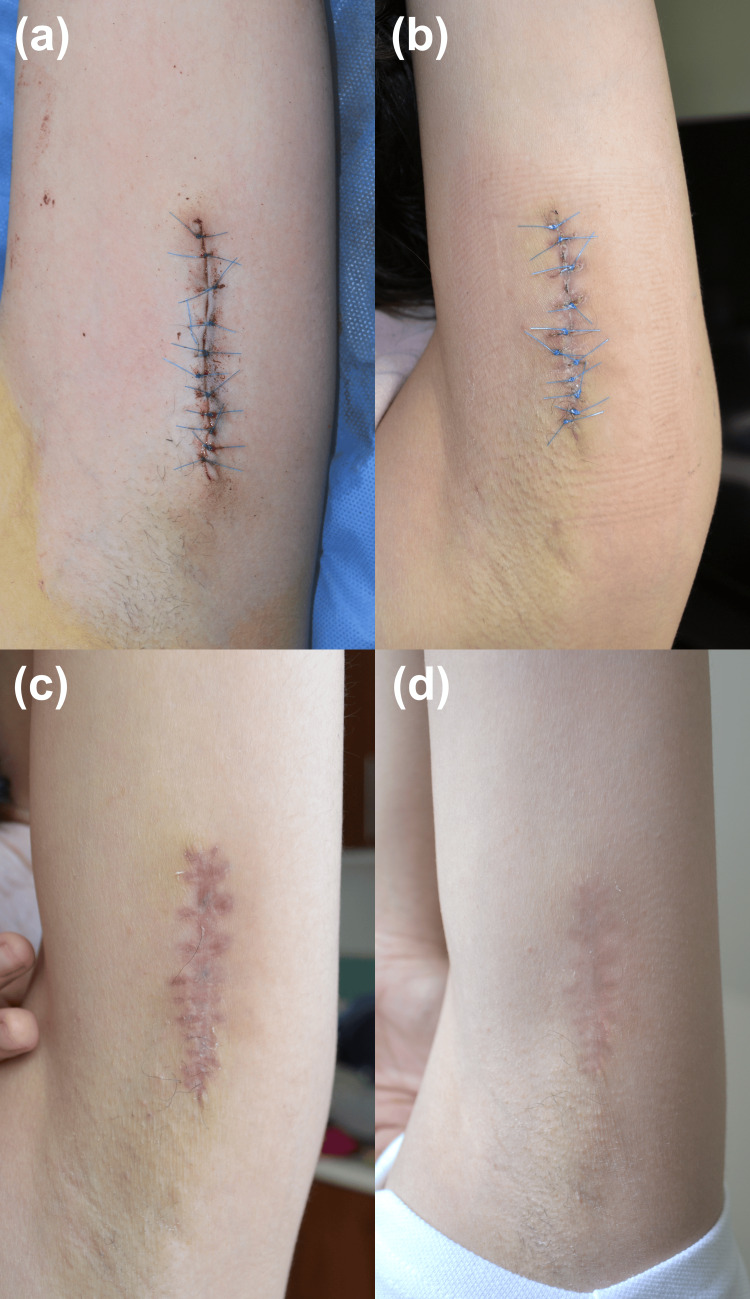
Post procedure follow-ups (a) Day 0 after excision, (b) Two weeks, (c) Four weeks, (d) Eight weeks.

The combination of its congenital onset, verrucous appearance, and linear distribution strongly suggested a clinical diagnosis of an isolated VEN. Given the lesion's size and the patient's desire for definitive removal, complete surgical excision was recommended. A full-thickness excision was performed, removing the entire lesion with 3-mm margins down to the subcutaneous tissue (Figure 2). Histopathological analysis of the specimen subsequently confirmed the diagnosis of VEN (Figure 3). At a two-month postoperative follow-up, the surgical site was well-healed with no evidence of recurrence, and the patient expressed satisfaction with the cosmetic outcome (Figure 4).

## Discussion

Epidermal nevi are benign hamartomatous lesions originating from the epidermis and its adnexal structures, composed of an abnormal proliferation of keratinocytes and occasionally other skin elements such as sebaceous glands, hair follicles, and sweat glands. VEN, the most common subtype, typically presents as linear, hyperpigmented, and often papillomatous or verrucous plaques that follow the lines of Blaschko on the skin [5,6].

Epidemiology

VENs are uncommon, with estimated population incidence ranging from 0.1% to 0.5% for keratinocytic nevi overall and specific rates of one to three per 1,000 for classic VEN [7,8]. Lesions most often present at birth or early childhood, although some may become clinically apparent later. Both genders are affected, with some reports suggesting a slight female predominance in special subtypes such as inflammatory linear verrucous epidermal nevi (ILVEN) [7,9]. VEN may occur sporadically, but rare familial or syndromic forms associated with multisystem involvement have been described [10,11].

Etiology

The pathogenesis of VEN is rooted in genetic mosaicism, with postzygotic somatic mutations in genes regulating epidermal proliferation and differentiation such as FGFR3, PIK3CA, HRAS, KRAS, AKT1, PTEN, NSDHL, KRT1, and KRT10. These mutations result in clonal cell populations that produce characteristic lesions following Blaschko’s lines. The specific mutation and timing during embryogenesis influence both the extent of cutaneous involvement and the risk of associated systemic features in epidermal nevus syndromes (ENS) [5,8,11]. ILVEN is associated with cytokine dysregulation, including elevated levels of interleukins and TNF-alpha [12]. Hormonal and environmental factors may also play a modifying role, especially in androgen-responsive nevi such as Becker’s nevus [10].

Histopathologic findings

VENs characteristically display hyperkeratosis, papillomatosis, and acanthosis with elongation of the rete ridges. Epidermolytic hyperkeratosis with vacuolar degeneration, parakeratosis, and acantholysis may be seen in certain variants, such as epidermolytic or acantholytic nevi. ILVEN is differentiated histologically by alternating parakeratosis with agranulosis and orthokeratosis with hypergranulosis, accompanied by psoriasiform features and a dermal inflammatory infiltrate. Some nevi present features that overlap with other dermatological conditions, emphasizing the need for clinicopathological correlation [5,13].

Clinical presentation

VEN presents as linear, well-demarcated, skin-colored to brown papules or plaques, with a verrucous, papillomatous, or velvety surface, typically following the lines of Blaschko and often persisting or thickening with age, as in the case of our patient. Lesions may be isolated or widespread and can affect any anatomical site including the trunk, limbs, neck, face, and rarely, mucosal surfaces. Systematized forms (nevus unius lateralis) and ENS may include neurologic, skeletal, or ocular anomalies. ILVEN is notable for pronounced pruritus, erythema, and chronic inflammation along a unilateral distribution, usually manifesting in infancy. Extensive involvement can cause significant cosmetic and psychosocial impact, and although rare, malignant transformation (e.g., basal cell carcinoma, squamous cell carcinoma, keratoacanthoma) has been reported in long-standing lesions [12-15].

Diagnosis

Diagnosis is primarily clinical, based on characteristic appearance and distribution along Blaschko’s lines. Dermoscopy, histopathologic evaluation, and occasionally genetic analysis support the diagnosis, help to differentiate among subtypes, and rule out mimickers such as linear and whorled nevoid hypermelanosis, lichen striatus, or mosaic eczema. In patients with extensive lesions or symptoms suggestive of ENS, a thorough systemic evaluation including neurologic, ophthalmologic, and skeletal assessment, plus laboratory and imaging studies, may be warranted [10,11].

Current treatments

Management of VEN is challenging, as there is no universally effective therapy and lesions are often recalcitrant with high recurrence potential. Topical agents, such as corticosteroids, retinoids, calcipotriol, 5-fluorouracil, and podophyllin, offer modest and usually transient improvement, especially in inflammatory forms like ILVEN. Cryotherapy is effective for small, localized lesions, achieving >75% clearance in up to 90% of cases, though hypopigmentation is a frequent side effect, particularly in darker skin. Carbon dioxide (CO₂) laser is useful for debulking extensive or facial lesions, providing good cosmetic results with careful technique, but may cause erythema, pigmentary changes, or scarring. Newer modalities such as Er:YAG laser provide improved tissue ablation with faster healing, less scarring, and fewer pigmentary changes; clearance rates are high, but recurrence (about 25%) can occur and repeat treatment is sometimes needed. Surgical excision, including full-thickness, staged, or tissue expansion techniques remains the gold standard for large or recalcitrant lesions and achieves long-term or permanent resolution, but scarring is inevitable, and large areas may require complex reconstruction. Photodynamic therapy (PDT), particularly when combined with pre-treatment modalities like superficial shaving or fractional micro-plasma radiofrequency, is emerging as a scar-sparing strategy for recalcitrant or widespread lesions, with case reports demonstrating long-lasting remission and good cosmetic outcomes. Indocyanine green-based PDT represents a well-tolerated, cost-effective option with minimal pain or scarring in isolated cases. Overall, treatment is tailored to lesion location, size, patient preference, and potential for recurrence or malignancy [12-15].

## Conclusions

VEN represent benign congenital hamartomas that pose primarily cosmetic concerns but require careful evaluation due to potential syndromic associations and rare malignant transformation risk. This case demonstrates the successful management of an isolated VEN in an 18-year-old patient through complete surgical excision. The lesion’s characteristic clinical presentation - linear distribution following Blaschko’s lines, progressive growth since birth, and verrucous hyperpigmented appearance - facilitated accurate clinical diagnosis, which was subsequently confirmed histopathologically. While multiple treatment modalities exist for VEN, including topical agents, cryotherapy, and various laser systems, these approaches often provide only modest and transient results with significant recurrence potential. In contrast, complete surgical excision remains the gold standard for definitive management, particularly for localized lesions like the one presented in this case. Despite the inevitable scarring associated with surgical intervention, this approach offers the most reliable long-term resolution. The successful outcome in this case, with complete healing and no recurrence at two-month follow-up, underscores the effectiveness of surgical excision for appropriately selected patients. Treatment selection should be individualized based on lesion characteristics, patient preferences, and the balance between therapeutic efficacy and potential complications. For small, isolated VENs causing cosmetic concern, complete surgical excision provides definitive cure with excellent patient satisfaction, as demonstrated in this case report.

## References

[1] Carbotti M, Coppola R, Graziano A, Verona Rinati M, Paolilli FL, Zanframundo S, Panasiti V (2016). Dermoscopy of verrucous epidermal nevus: large brown circles as a novel feature for diagnosis. Int J Dermatol.

[2] Paller AS, Syder AJ, Chan YM, Yu QC, Hutton E, Tadini G, Fuchs E (1994). Genetic and clinical mosaicism in a type of epidermal nevus. N Engl J Med.

[3] Vidaurri-de la Cruz H, Tamayo-Sánchez L, Durán-McKinster C, de la Luz Orozco-Covarrubias M, Ruiz-Maldonado R (2004). Epidermal nevus syndromes: clinical findings in 35 patients. Pediatr Dermatol.

[4] Panagiotopoulos A, Chasapi V, Nikolaou V, Stavropoulos PG, Kafouros K, Petridis A, Katsambas A (2009). Assessment of cryotherapy for the treatment of verrucous epidermal naevi. Acta Derm Venereol.

[5] Bolognia JL, Orlow SJ, Glick SA (1994). Lines of Blaschko. J Am Acad Dermatol.

[6] Park JH, Hwang ES, Kim SN, Kye YC (2004). Er:YAG laser treatment of verrucous epidermal nevi. Dermatol Surg.

[7] Peng L, Li Y, Jiang Z, Luo Z (2023). Superficial shaving combined with photodynamic therapy for treating verrucous epidermal nevi: a case report. Photodiagnosis Photodyn Ther.

[8] Garcias-Ladaria J, Cuadrado Rosón M, Pascual-López M (2018). Epidermal nevi and related syndromes -- Part 1: keratinocytic nevi. Actas Dermosifiliogr (Engl Ed).

[9] Zheng X, He S, Li Q (2018). Successful treatment of verrucous epidermal nevus with fractional micro-plasma radio-frequency technology and photodynamic therapy. J Cosmet Laser Ther.

[10] Brandling-Bennett HA, Morel KD (2010). Epidermal nevi. Pediatr Clin North Am.

[11] Asch S, Sugarman JL (2018). Epidermal nevus syndromes: new insights into whorls and swirls. Pediatr Dermatol.

[12] Lee BJ, Mancini AJ, Renucci J, Paller AS, Bauer BS (2001). Full-thickness surgical excision for the treatment of inflammatory linear verrucous epidermal nevus. Ann Plast Surg.

[13] Kim TI, Jeong KH, Shin MK (2015). Verrucous epidermal nevus (VEN) successfully treated with indocyanine green (ICG) photodynamic therapy (PDT). JAAD Case Rep.

[14] Lapidoth M, Israeli H, Ben Amitai D, Halachmi S (2013). Treatment of verrucous epidermal nevus: experience with 71 cases. Dermatology.

[15] Borzecki A, Strus-Rosińska B, Raszewska-Famielec M, Sajdak-Wojtaluk A, Pilat P (2016). Linear verrucous epidermal nevi-effects of carbon dioxide laser therapy. J Cosmet Laser Ther.

